# Dentistry Fifty Years Ago

**Published:** 1912-09-15

**Authors:** J. P. Root


### DENTISTRY FIFTY YEARS AGO.

From the Dental Cosmos, 1860.

BY J. P. ROOT, D.D.S.

After testing for one year the qualities of the *Cosmos,* Jones and White, the publishers, decided to observe, compare, reflect, and record the doings of the dentists of the

United States, and with the able assistance of the original editorial staff continue the publication of the *Cosmos.*

J. D. White started Vol. II by observing in the "Practical Hints" department that the absorption of dentine in roots of permanent teeth had not, so far as "we" are aware of, received its just attention. He gave the subject the attention deserved, but failed to enlighten the public as to the cause.

Under the title of "Empiricism" one D. T. L. had a scathing article, censuring the patenting of "nearly everything we use in our art," and frequently by men who were not the discoverers, as these "scorned to clothe themselves with professional infamy for the sake of securing the almighty dollar." Some people were just as bitter against the patentee then as now. D. T. L. cites a case recently published in *Cosmos* regarding an original method of curing toothache, and wonders why it was not patented as the author said, "If I find the toothache proceeds from an exposed nerve, I immediately advise filling, without any preparatory treatment, except the application of creosote for a moment." The author says he advises immediate filling, does not mention the fact he fills, nor does he mention his hiding place if he did.

The serious question regarding the influence of saliva upon the human system, especially the teeth, has always been a favorite subject with editors of the *Cosmos.* Dr. McQuillen, of the editorial staff, devotes seven pages of fine print to establishing facts regarding this secretion. It reads as interestingly, and is nearly, if not quite, as learned, as editorials on this subject printed in the *Cosmos* today, and throws about as much light on the subject. Dr. McQuillen did not mention any influence saliva might have upon the indications of child birth. This question was settled forty years later at a Chicago dental meeting by one of his successors.

Dr. P. G. C. Hunt (father of George Edwin), at a meeting in Indianapolis, stated, "Dentistry is said to be a specialty of medicine." Some still so state this questionable fact, and probably fifty years from now when some intrepid fool editor reviews the dental literature of today, he will probably notice that the movement is still going on, if not spreading. Dr. Hunt indicates by some prophylactic remarks, where George Edwin received his writing blood such as entitles him to edit the famous or otherwise journal he at present bosses. He said, "With the dentist in full practice, it is a matter of everyday occurrence to see mouths so replete with salivary calculus and accumulated filth as to leave no doubt in the mind of anyone who reasons from cause to effect that to this cause, in many cases, might with truth be referred disturbances more or less serious in the alimentary canal and lungs."

Dr. White, in attending to the "premature extraction of deciduous teeth for apparent irregularities," gives sound advice, just as good today, and perhaps as muchly needed. He says, "We do not extract deciduous teeth, except under extraordinary circumstances, especially the superior canines." What business has a dentist to interfere with the half-completed evolutions of nature in her most beautiful and complicated developments. There are but two answers: Lack of moral responsibility, or ignorance.

J. Foster Flagg makes some claims which he admits may be arrogant and untenable. He told the American Dental Association members some facts regarding "Our Calling." He said: "*It claims from its practitioners a degree of combined moral, physical and general intellectual attainment, equal to, if not greater, than that of any other calling.* He said: "The dentist should be governed by the highest moral influences in his intercourse with patients." He cites as examples: "That the clergyman has thrown around him the sustaining aid of religion, of position, of association; around the physician are thrown the barriers of the patient's home, the presence of members of the family," etc. While the dentist evidently lacks the sustaining aid of religion and the barriers of the physician, so must necessarily be of a higher moral character than they.

In August, 1860, C. P. Fitch read a paper before the American Dental Association on "Extraction of Teeth." He said: "That too many teeth are extracted, even at the present time, is a fact too patent to be denied. We insist no tooth should be extracted until remedial agencies have been exhausted in vain for its preservation. Preserving treatment and fang filling have done much in dental achievement. Some dentists gain quite an enviable notoriety as tooth-pullers. They are metamorphosed into walking, breathing, machines of torture, rioting in bone, flesh and blood, and moved by an energy and zeal which if properly directed would be worthy of imitation."

At the same meeting Geo. T. Barker said, "There is a demand from our patients upon us for an improved method of treatment, some agent that will act as a palliative, while it will not affect unfavorably the sensitive pulp, or destroy the surrounding tooth-substance; an agent that will give our patients a confidence to come to us in the earliest stages of carious action"—(said necessity still exists). He demanded "Educated practitioners, both in literature and science, with minds that can make fingers work out thoughts of the brain; not merely a scientific knowledge or a dextrous workmanship." (We are still hunting for this combination.) Dr. Barker must have been on the original Reorganization Committee of the National, for he ended his remarks with, "A short reference to a demand of the age which has just been met. I refer to a National Dental Association, upon a strictly representative basis, which will, I feel assured, redound to the credit of the profession, stimulating associated effort in local societies, elevating the standard of dental science, creating upon every side a liberality of sentiment that will win for our art the title of a liberal, elevated, and scientific profession, and eventually the true demands upon dental science will be met." Fifty-two years later (today), the same demand exists, and judging by the procrastinatory methods used, the same demand will be upon our descendants fifty years hence.

William H. Atkinson, at the National meeting said: "Let each one freely open his mind, and not be frightened at his own shadow, but persistently keep it opened till it is filled with something to give out, then truthfully and plainly project it. Let us have freedom from dignity and restraint and have fraternity and unity of feeling in the interchange of sentiments. At this meeting, held at Washington, D. C., July 31, 1860, Dr. Allport, of Chicago, presided; J. Taft was secretary; twenty-three delegates were present at the opening. A constitution and by-laws were adopted. The officers-elect were: President, W. H. Atkinson, Cleveland; First Vice-President, J. B. Gibbs, Washington, D. C.; Second Vice-President, F. Y. Clark, Savannah, Ga.; Recording Secretary, J. Taft, Cincinnati; Corresponding Secretary, W. Muir Rogers, Shelbyville, Ky. Among the resolutions passed was one "That the American Dental Association respectfully recommends to dental colleges the necessity of their being more stringent in requiring of matriculants a higher order of general literary and scientific education, and a rigid enforcement of their standard requirements for graduation." This reads as if coming from our present National Board of Dental Examiners, directed to the National Association of Dental Faculties, and probably received as much attention as if such was the case.

The American Dental Convention met at Saratoga Springs, August 7, 1860. The officers-elect were: President, T. L. Buckinham, Philadelphia; Vice-President, B. A. Rodrigues, Charleston; Recording Secretary, J. B. Wetherbee, Boston; Treasurer, A. N. Priest, Utica, N. Y. Most of this session was devoted to discussing artificial plates and capping exposed pulps. Dr. Dwinell stated, when he treated inflamed pulps verging on the supperative stage, he applied leeches locally, and salts constitutionally. Certainly any self-respecting pulp would behave after this treatment.

In the editorial department of the September, 1860, issue, J. D. White gives J. Taft some free advertising regarding some doubtful facts in "Taft's Operative Dentistry." This was a continuation of a former editorial not finished "for want of space," but judging from its tenor, J. D. White ran out of vitriol. Reviewing the chapter on "Irregularities," he says, "If one cannot teach properly, it is better not to teach at all." This was meant for J. Taft's edification. The chapter on "Atrophy" is attended to "as of not much practical value, and will not receive attention." J. D. was some sarcastic when he said, "The article on Exostosis is very good and the author reminds the reader very frequently that a tooth affected by it is very difficult to extract." This editorial was continued—no reason given but probably so as to allow J. Taft time to recover or withdraw his text-book from the market. Judging from most of J. D. White's editorials they must have been written before breakfast, at a time known as "The dark gray dawn of an early morn," a time not propitious for frivolous remarks.—*Western Dental Journal.*

